# Expression of *OsHARBI1-1* enhances the tolerance of *Arabidopsis thaliana* to cadmium

**DOI:** 10.1186/s12870-023-04540-0

**Published:** 2023-11-11

**Authors:** Nan Jiang, Yang Shi, Mingyu Li, Zhiye Du, Ji Chen, Wenjun Jiang, Yanyan Huang, Min Zhong, Ju Yang, Binhua Hu, Jin Huang

**Affiliations:** 1 College of Ecology and Environment, Chengdu University of Technology, Chengdu, 610059 China; 2 College of Agronomy, Sichuan Agricultural University, Chengdu, 611130 China; 3 State Key Laboratory of Crop Gene Exploration and Utilization in Southwest China, Sichuan Agricultural University, Chengdu, 611130 China; 4 Institute of Biotechnology and Nuclear Technology, Sichuan Academy of Agricultural Sciences, Chengdu, 610066 China

**Keywords:** HARBI1, Cadmium tolerance, Rice, *Arabidopsis thaliana*, Yeast, Antioxidant enzyme activities

## Abstract

**Background:**

As one of the major food crops in the world, rice is vulnerable to cadmium (Cd) pollution. Understanding of the molecular mechanisms of Cd uptake, transport and detoxification in rice is essential for the breeding of low-Cd rice. However, the molecular mechanisms underlying the response of rice to Cd stress remains to be further clarified.

**Results:**

In this study, a novel Cd-responsive gene *OsHARBI1-1* was identified in the rice genome and its expression pattern and function were characterized. Bioinformatics analysis showed that the promoter region of *OsHARBI1-1* had multiple cis-acting elements in response to phytohormones and stress, and the expression of *OsHARBI1-1* was induced by phytohormones. OsHARBI1-1 protein was targeted to the nucleus. qRT-PCR analysis results showed that the expression of *OsHARBI1-1* in the roots was repressed while the expression in the shoots was increased under Cd stress. Heterologous expression of *OsHARBI1-1* in yeast conferred tolerance to Cd and reduced Cd content in the cells. Meanwhile, the expression of *OsHARBI1-1* in *Arabidopsis thaliana* (*A. thaliana*) enhanced the tolerance of *A. thaliana* to Cd stress. In addition, compared with the wild type plants, the POD activity of transgenic plants was increased, while the SOD and CAT activities were decreased. Interestingly, the accumulation of Cd in the roots of *A. thaliana* expressing *OsHARBI1-1* was significantly increased, whereas the Cd accumulation in the shoots was slightly decreased. Compared to the WT plants, the expression of genes related to Cd absorption and chelation was upregulated in transgenic *A. thaliana* under Cd stress, while the expression of genes responsible for the translocation of Cd from the roots to the shoots was downregulated. Moreover, the expression of phytohormone-related genes was significantly influenced by the expression of *OsHARBI1-1* with and without Cd treatment.

**Conclusions:**

Findings of this study suggest that *OsHARBI1-1* might play a role in the response of plants to Cd response by affecting antioxidant enzyme activities, Cd chelation, absorption and transport, and phytohormone homeostasis and signaling.

**Supplementary Information:**

The online version contains supplementary material available at 10.1186/s12870-023-04540-0.

## Background

Cadmium (Cd), as a toxic heavy metal, is neither nutritive nor essential to plants. When absorbed by plants, Cd disrupts series of physiological processes such as photosynthesis, cell division and enzyme activities, leading to stunted growth, leaf chlorosis and yield losses [1–5]. Meanwhile, Cd is accumulated in the edible parts of plants and thus easily enters the human body through the food chain [6, 7]. In this context, rice as one of the most important staple food crops, is the main source of dietary Cd intake [8]. Given this, Cd poses a huge potential threat to human health, especially for Asians who consume rice as a staple food [8–10]. Thus, the problem of how to reduce the Cd content in rice grains has become a research hot spot in recent years.

In response to Cd stress, rice has evolved intricate signaling and defense pathways. Studies have showed the involvement of metal transporters including heavy metal-associated domain (HMA) family, metal tolerance proteins (MTP) family and natural resistance-associated macrophage protein (NRAMP) family in the uptake and transport of Cd in rice. Meanwhile, Cd stress triggers a signaling cascade mediated by various transcription factors, such as MYB family and WRKY family, consequently activating the expression of antioxidant enzymes in rice to improve the Cd tolerance. Furthermore, metal chelators including phytochelatins, metallothioneins and glutathione also play a vital role in reducing Cd toxicity to rice by effectively chelating Cd ions [11–16]. Apart from the aforementioned genes and mechanisms, a number of, Cd-tolerant or -responsive genes, such as *OsSNAC1*, *OsJAZ9*, *OsNPR4* and *OsHIPP42* also have been identified via yeast-based cDNA library survival screening or bioinformatics analyses [17, 18]. However, the molecular mechanisms underlying plant responses to Cd stress remain to be further elucidated.

In this study, a Cd-responsive gene of unknown function encoding a Harbinger Transposase Derived 1 (HARBI1) protein in rice was identified and named *OsHARBI1-1* (Locus: *LOC_Os01g40070*). *HARBI1* genes belong to DDE_Tnp_4 subfamily within DDE superfamily and originate from domesticated of PIF/Harbinger transposases, which were first discovered and named by Kapitonov in animals [19]. HARBI1 proteins contain conserved DDE catalytic domains that bind divalent metal ions and catalyze DNA cleavage [20]. In plants, *HARBI1* genes have developed new cellular functions to benefit the host [21]. For example, certain *HARBI1* genes in rice and *Arabidopsis thaliana* (*A. thaliana*) have been reported to play critical roles in regulating plant growth and development [21–24]. However, limited research has been conducted on the role of *HARBI1* genes in regulating stress response in plants. A previous study has shown that over-expression of *MdHARBI1* enhances thermo-tolerance in tomato (*Solanum lycopersicum*) [25]. In *A. thaliana*, as a pair of paralog genes, *AtHARBII-1* and *AtHARBI1-2* are strongly induced by abiotic stress [26, 27]. These findings suggest that *HARBI1* genes in plants are involved in response to abiotic stresses. Nevertheless, it remains unclear whether *HARBI1* genes are also involved in the response to heavy metal stress in plants.

The objective of this study is to reveal the functions of *OsHARBI1-1* in plants exposed to Cd stress. Therefore, *OsHARBI1-1* gene was introduced into yeast (*Saccharomyces cerevisiae*) and *A. thaliana*, and the tolerance of transgenic yeast and *A. thaliana* to Cd stress was analyzed. In addition, the antioxidant enzyme activities and the Cd content in transgenic *A. thaliana* were also determined to further explore the molecular function of *OsHARBI1-1* under Cd stress. In this study, *OsHARBI1-1* was identified as a highly responsive gene to Cd stress in rice. In addition, the results showed that the expression of *OsHARBI1-1* significantly enhanced the Cd tolerance of yeast and *A. thaliana*. The findings shed light on the molecular mechanisms of Cd resistance in rice and provide a potential genetic resource for the phytoremediation of Cd contamination.

## Results

### Bioinformatics analysis of 33 *OsHARBI1* genes in rice genome

**Fig. 1:**
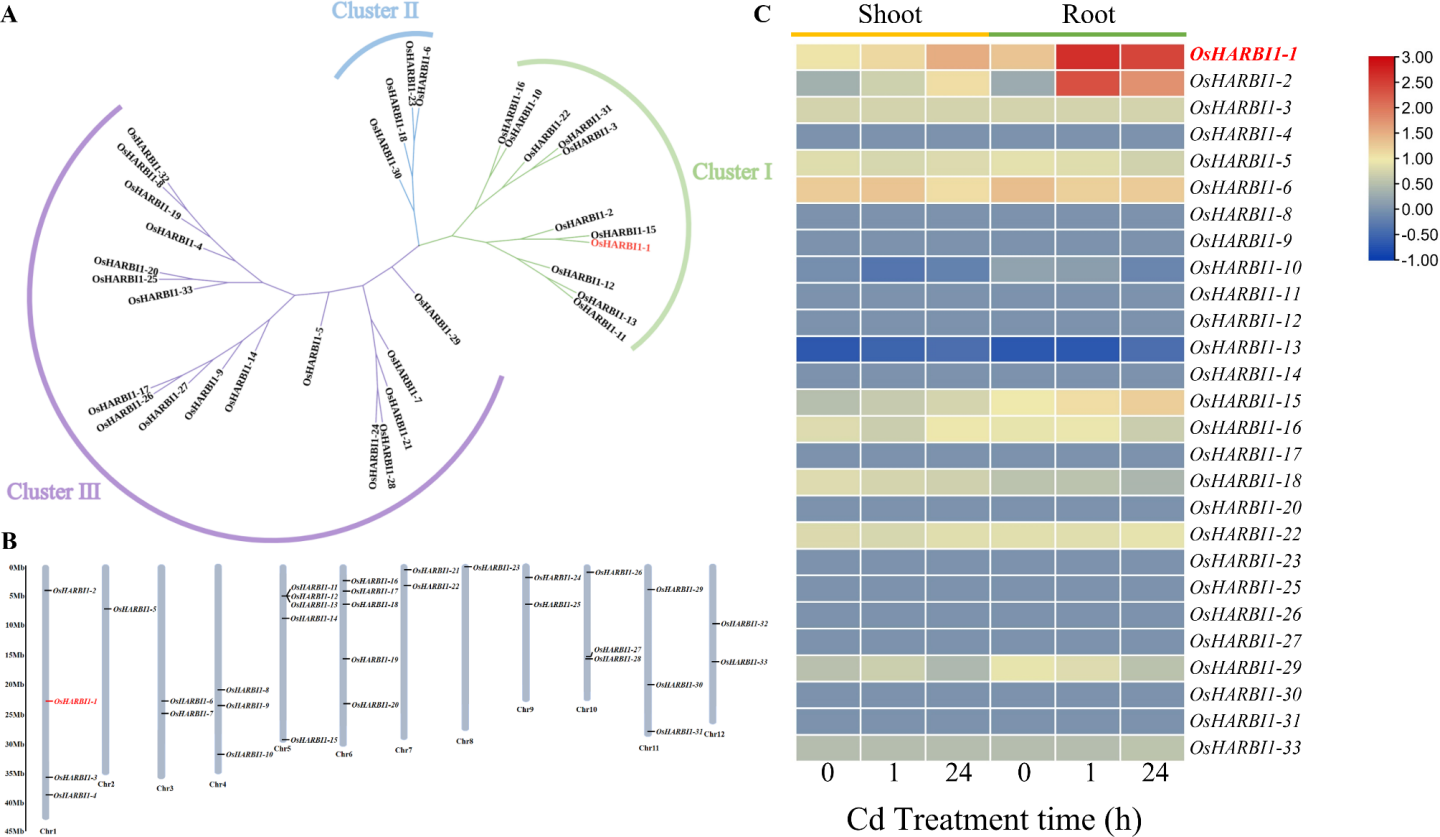
Bioinformatics analysis of OsHARBI1 protein family. **A** Phylogenetic tree of HARBI1 proteins in rice. Phylogenetic analysis was constructed according to the DDE domains of HARBI1 proteins by using the Clustal X algorithm. Three clusters were classified and each cluster was presented with a different color. **B** Chromosomal location of *HARBI1* genes in rice. The 33 *HARBI1* genes were widely distributed on 12 chromosomes. The gene chromosome location diagram was generated using TBtools software. **C** Expression profiles of rice *HARBI1* genes under Cd stress. The heat map was generated using the data retrieved from Rice Expression Database with the assiatance of TBtools.

OsHARBI1 protein is annotated as “Putative nuclease HARBI1-like” (InterPro accession: IPR045249), suggesting that it may be a “domesticated” protein from Harbinger transposase. To date, HARBI1 has not been functionally analyzed in rice yet. In order to further understand the functional characteristics of OsHARBI1, bioinformatics analysis of OsHARBI1 family was performed. The HARBI1 protein family in rice was identified by using the DDE domain. A total of 33 *HARBI1* genes were identified in the rice genome (Table S2), and these proteins were divided into three subgroups through phylogenetic analysis (Fig. 1A). Chromosomal localization analysis showed that *OsHARBI1* genes were dispersed across all chromosomes of rice, and their names were assigned from *OsHARBI1-1* to *OsHARBI1-33* according to their distribution on the chromosomes (Fig. 1B). To better understand the functions of *OsHARBI1* genes, their expression patterns under Cd stress were analyzed using Rice Expression Database, while the cis-elements in the promoter region of *HARBI1* genes were investigated using PlantCARE. The expression analysis data showed that the expression of only 2 (*OsHARBI1-1* and *OsHARBI1-2*) of 33 *HARBI1* genes was highly induced by Cd stress (Fig. 1C). In addition, cis-acting element analysis revealed the presence of various stress and hormone responsive cis-elements in the promoter regions of *OsHARBI1* genes, including those associated with light responsiveness, low-temperature responsiveness, drought inducibility, anaerobic induction, auxin responsiveness, abscisic acid (ABA) responsiveness, gibberellin (GA) responsiveness and methyl jasmonate (MeJA) responsiveness (Fig. S1). The results of cis-acting element analysis suggest that the potential regulation of *OsHARBI1-1* expression by phytohormones. Therefore, the expression pattern of *OsHARBI1-1* under phytohormone treatments was analyzed using Rice Expression Profile Database (RiceXPro). The results showed that the expression of *OsHARBI1-1* in the rice shoots remained unaffected by phytohormones treatments. Interestingly, in the roots, when treated with ABA treatment for 3 and 6 h, as well as JA (jasmonic acid) treatment for 1 h, 3 and 6 h, the expression of *OsHARBI1-1* was significantly upregulated (Fig. S2).

### The expression of *OsHARBI1-1* was induced by Cd stress

**Fig. 2:**
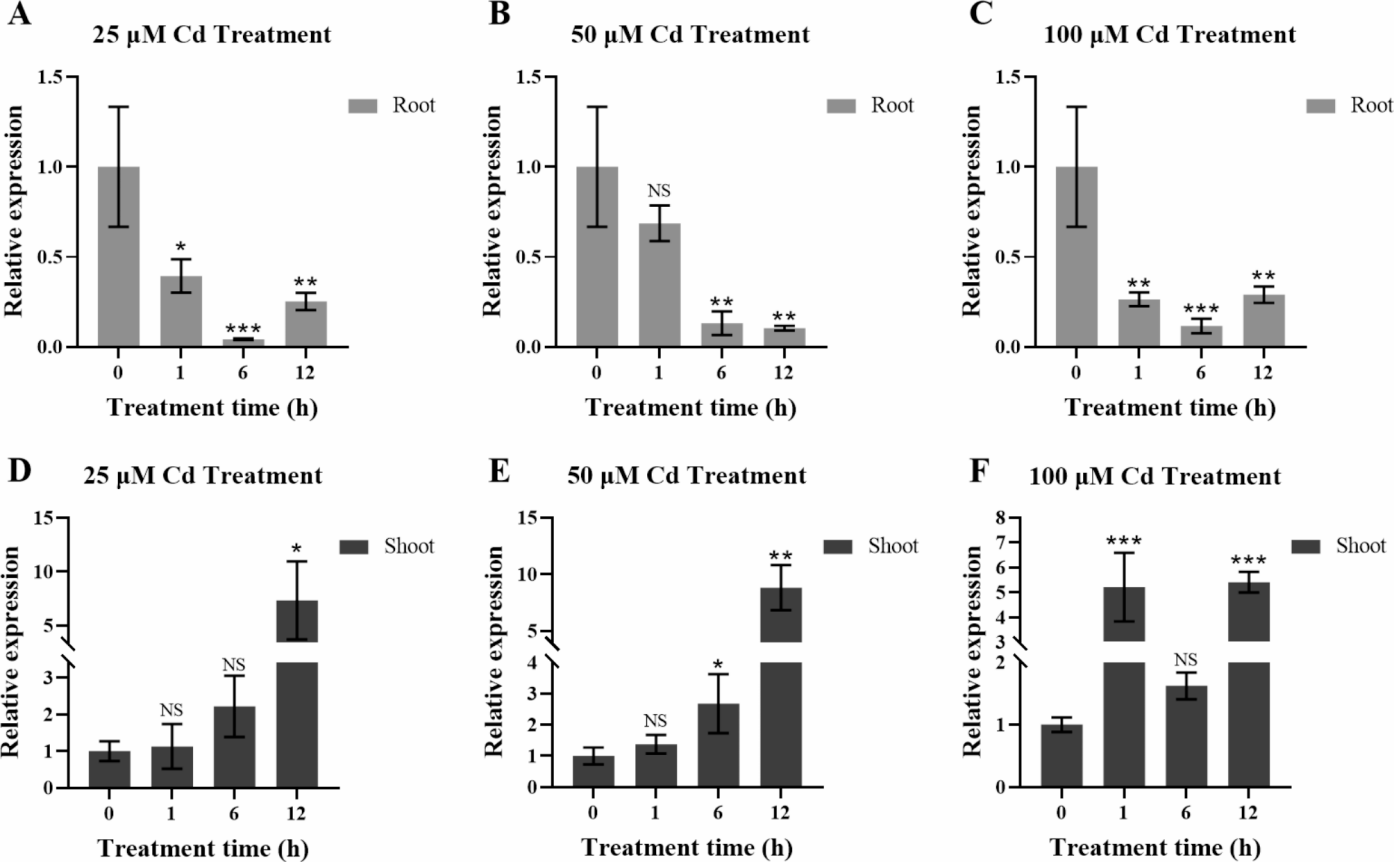
Expression of *OsHARBI1-1* when treated with differently concentrated Cd. The expression patterns of *OsHARBI1-1* in the shoots (**A-C**) and roots (**D-F**) were measured under 25 (A and D), 50 (B and E) or 100 (C and F) µM Cd treatment for 1, 6 and 12 h, respectively. Results were calculated using 2^−ΔΔCT^ method with *Ubiquitin* as internal reference gene. Shown are mean ± standard deviation (SD) from three biological replicates. Asterisks indicate significant differences (Student’s t-test: NS means non-significant; *P < 0.05; **P < 0.01; ***P < 0.001)

To investigate the expression pattern of *OsHARBI1-1* under Cd treatment, the expression levels were analyzed by using qRT-PCR. The results showed that the expression levels of *OsHARBI1-1* in the roots were repressed by Cd treatment (Fig. 2A-C). Conversely, the expression levels of *OsHARBI1-1* in the shoots were increased at all concentrations of Cd treatment (Fig. 2D-F). Notably, for short-term (1 h) Cd treatment, unlike the expression of *OsHARBI1-1* which was not significantly affected under 25 and 50 µM Cd treatment, the expression of *OsHARBI1-1* was strongly induced by 100 µM Cd treatment (Fig. 2D-F). These data indicate that *OsHARBI1-1* was a typical Cd-responsive gene in rice.

### Expression of *OsHARBI1-1* conferred enhanced Cd tolerance to yeast

**Fig. 3:**
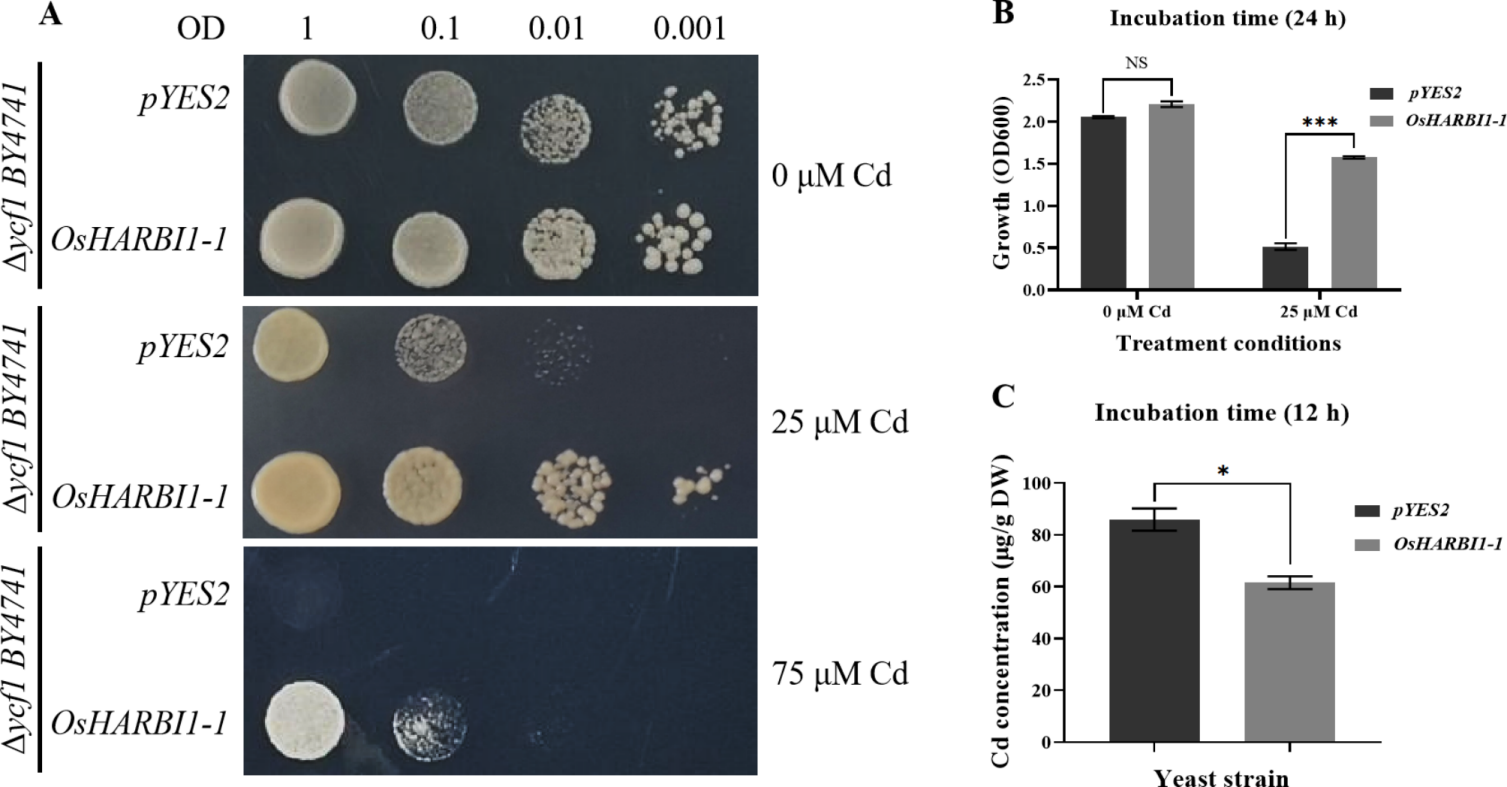
Functional analysis of *OsHARBI1-1* in yeast. **A** The effects of *OsHARBI1-1* on yeast resistance to Cd stress. Cd-sensitive mutant *Δycf1* carrying *OsHARBI1-1* cDNA or empty vector *pYES2* was treated with different concentrations (0, 25 and 75 µM) of Cd for three days. **B** The growth curves assay of yeast cells when treated with Cd. The yeast strain of *Δycf1* transformed with the empty vector *pYES2* or *OsHARBI1-1* was grown in SD-Ura liquid medium supplemented with 25 µM Cd, and cell densities were determined at 24 h. **C** The Cd content in the yeast cells. The yeast strains of *Δycf1* transformed with the empty vector *pYES2* or *OsHARBI1-1* were grown in SD-Ura liquid medium supplemented with 10 µM Cd for 12 h, and the Cd contents were detected using an inductively coupled plasma atomic absorption spectrometer (ICP-AES). Shown are mean ± standard deviation (SD) from three biological replicates. Asterisks indicate significant differences (Student’s t-test: NS means non-significant; *P < 0.05; ***P < 0.001)

Yeast is a model organism which is widely used for functional characterization of plant genes [28]. Yeast transformed with *OsHARBI1-1* was employed to investigate the function of *OsHARBI1-1*. The results showed that *OsHARBI1-1* improved the tolerance of Cd in yeast (Fig. 3A and B). Moreover, decreased Cd accumulation was detected in the yeast cells containing *OsHARBI1-1* compared to that of the control (Fig. 3C). These findings further suggest that *OsHARBI1-1* may play important role in Cd tolerance in rice.

### OsHARBI1-1 proteins were localized in the nucleus

**Fig. 4:**
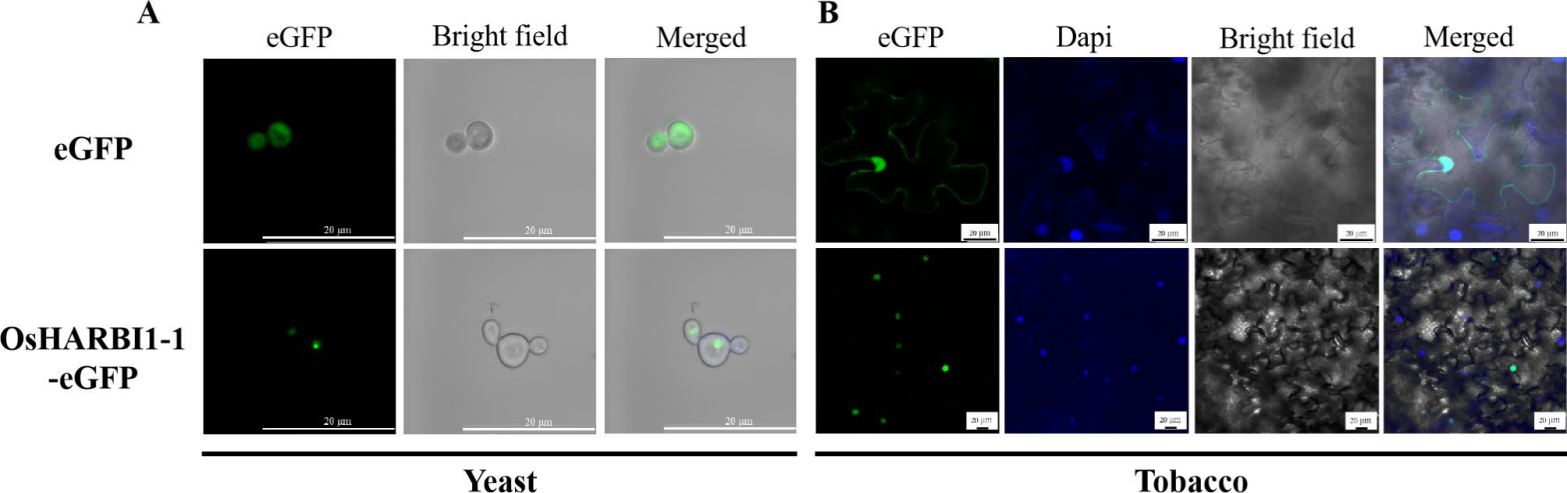
Subcellular localization of OsHARBI1-1 protein. **A** Subcellular localization of OsHARBI1-1 protein in yeast cells. eGFP and OsHARBI1-1-eGFP were induced in SD-Ura (galactose) for 6 h, and subcellular localization was observed under fluorescence microscope. **B** Subcellular localization of OsHARBI1-1 protein in tobacco epidermic cells. eGFP fluorescence was shown in green and the nucleus was blue with DAPI staining. Bar = 20 μm

The subcellular localization of proteins is often intimately linked to their biological functions [22, 25]. To further analyze the function of OsHARBI1-1 protein, the subcellular localization of OsHARBI1-1 protein in yeast cells and tobacco leave cells was analyzed respectively. The results clearly showed that the GFP signal of OsHARBI1-1-eGFP fusion proteins was observed in the nucleus. By contrast, the fluorescence signal generated by free eGFP was observed in the whole cells without specificity (Fig. 4A). In tobacco cells, the fluorescence signal emitted by OsHARBI1-1-GFP proteins was exclusively co-localized with that emitted by 4’, 6-diamidino-2-phenylindole (DAPI), whilst the fluorescence generated by free GFP proteins was diffusely throughout the tobacco cells (Fig. 4B). Therefore, a nucleus localization was suggested for OsHARBI1-1 protein in both yeast or plant cells.

### Expression of *OsHARBI1-1* conferred enhanced Cd tolerance to *A. thaliana*

**Fig. 5:**
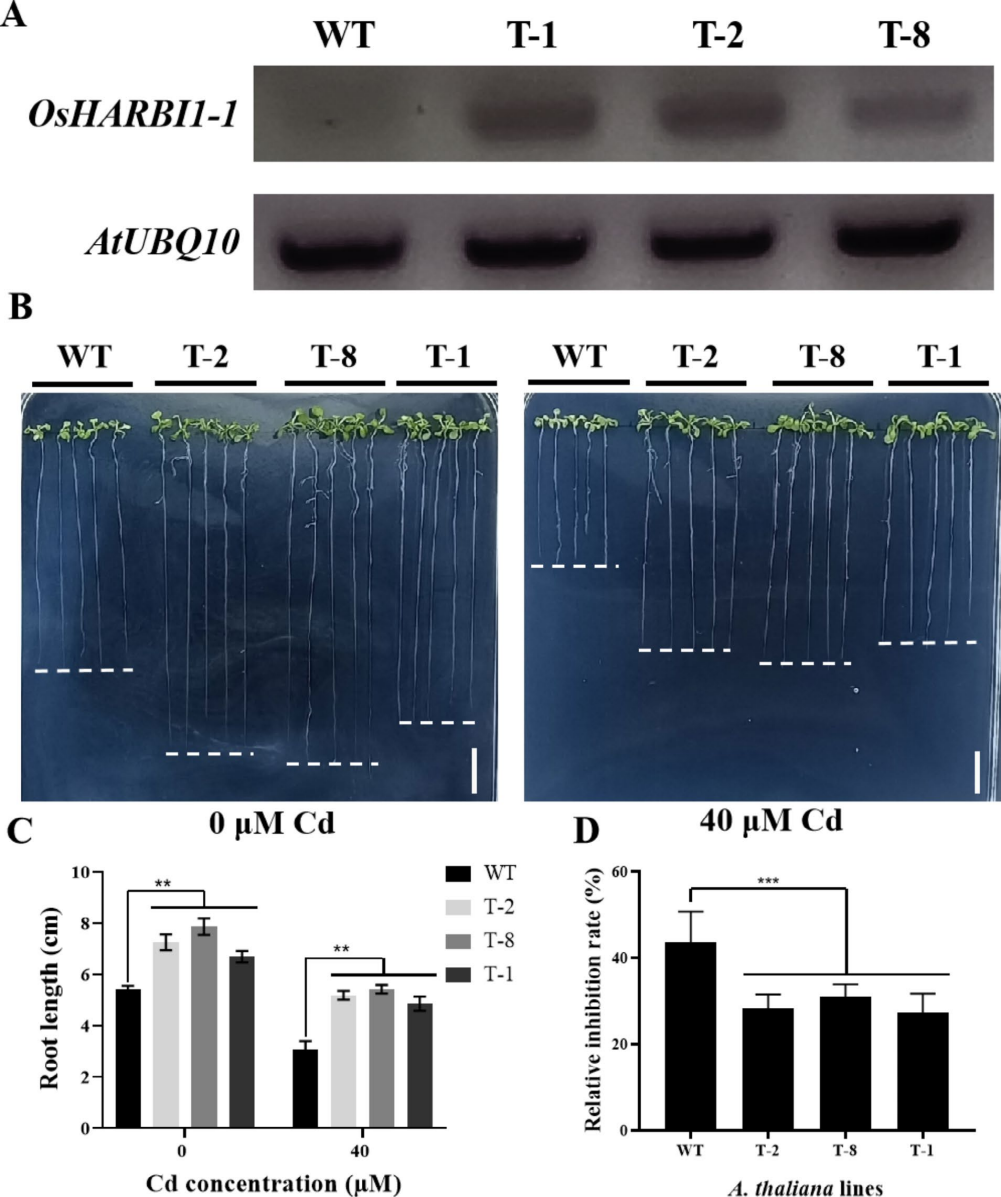
Phenotypic analysis of transgenic *A. thaliana* lines under Cd treatment. **A** Expression of *OsHARBI1-1* was analyzed in transgenic *A. thaliana* lines (T-2, T-8 and T-1) by PCR. *AtUBQ10* gene was used as an internal control. The full images could be found in the supplement Fig. S3. **B** The growth of *OsHARBI1-1* transgenic and control lines under Cd treatment. WT and transgenic plants were grown on vertical 1/2 MS solid agar plates supplemented with 0 µM Cd and 40 µM Cd, respectively. The seedlings were grown for 14 days. Bars = 1 cm. **C** The root length of WT and transgenic lines under Cd treatment. Plants were grown under the same conditions as (**A**). **D** The relative inhibition rate of root length of WT and transgenic lines under Cd treatment. Plants were grown under the same conditions as (**A**). Each experiment was conducted with three biological replicates. Shown are mean ± standard deviation (SD) from three biological replicates. Asterisks indicate significant differences between transgenic *A. thaliana* lines and WT (Student’s t-test: **P < 0.01; ***P < 0.001)

To gain further insight into the function of *OsHARBI1-1* under Cd stress, *OsHARBI1-1* transgenic *A. thaliana* plants were generated. The transgenic lines were confirmed using PCR (Fig. 5A and Fig. S2). To analyze the response of transgenic *A. thaliana* under Cd treatment, both WT and transgenic plants (T-2, T-8 and T-1) were grown on 1/2 MS media containing Cd (0 and 40 µM Cd respectively). After 2 weeks, the length of the Cd-treated plants was evaluated. The results showed that the expression of *OsHARBI1-1* promoted root growth of *A. thaliana* without Cd treatment (Fig. 5B and C). Moreover, when treated with 40 µM Cd, root growth of the WT line was inhibited by 44%, while the roots of transgenic lines showed only 27–30% inhibition (Fig. 5D). These findings showed that *OsHARBI1-1* enhanced the tolerance of *A. thaliana* to Cd.

### Expression of *OsHARBI1-1* significantly affected the accumulation and translocation of Cd, and altered antioxidant enzyme activities in *A. thaliana*

**Fig. 6:**
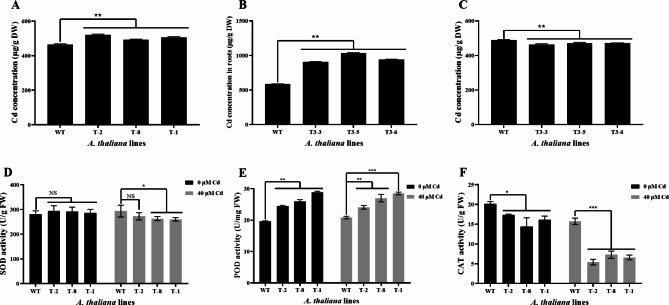
Analysis of Cd contents and antioxidant enzyme activities in WT and transgenic *A. thaliana* lines. The Cd content in whole seedlings (**A**), the roots (**B**) and the shoots (**C**) of 2-week-old WT and transgenic *A. thaliana* lines after 40 µM Cd treatment were measured. Antioxidant enzyme activities of SOD (**D**), POD (**E**) and CAT (**F**) were measured in 2-week-old *A. thaliana* plants under 0 or 40 µM Cd treatment. Each experiment was conducted with three biological replicates. Shown are mean ± standard deviation (SD) from three biological replicates. Asterisks indicate significant differences between transgenic *A. thaliana* lines and WT (Student’s t-test: NS means non-significant; *P < 0.05, **P < 0.01, ***P < 0.001)

To evaluate the effect of *OsHARBI1-1* on Cd accumulation and translocation, the Cd contents in the roots, shoots and whole seedlings of WT and transgenic lines were determined using ICP-MS after 2 weeks of 40 µM Cd treatment. The results showed that compared with WT plants, expression of *OsHARBI1-1* significantly increased the Cd content in the roots and whole seedlings, while the Cd content was slightly decreased in the shoots of the transgenic plants (Fig. 6A-C).

To further explore whether *OsHARBI1-1* affects the response of antioxidant enzyme system in transgenic *A. thaliana* under Cd stress, the activities of three antioxidant enzymes (SOD, POD and CAT) were determined. The results showed that, compared with WT plants, the SOD and CAT activities of transgenic *A. thaliana* lines were significantly decreased, and the POD activities were significantly increased under Cd stress (Fig. 6D-F). Interestingly, compared with WT, the SOD activities of transgenic *A. thaliana* lines were increased and CAT activities were decreased under 0 µM Cd treatment, which exhibited a similar trend to the Cd stress (Fig. 6D and F). These findings imply that *OsHARBI1-1* affects the intracellular antioxidant enzyme activity in *A. thaliana*, which may further affect the tolerance of *A. thaliana* to Cd by affecting the ROS levels.

### Expression of *OsHARBI1-1* affected the expression of genes related with chelation, absorption and transport of Cd in *A. thaliana* under Cd stress

In this study, expression of *OsHARBI1-1* in *A. thaliana* resulted in a significant increase in Cd content in the roots but a decrease in Cd content in the shoots. To investigate how *OsHARBI1-1* affected Cd concentration in *A. thaliana*, the expression of genes related with chelation, absorption and transport of Cd was analyzed using qRT-PCR. These genes included *AtHMA2/3/4* (heavy metal associated protein 2/3/4), *AtIRT1* (Fe-regulated transporter 1), *AtNramp1/3/4* (natural resistance-associated macrophage protein 1/3/4), *AtPDR8* (pleiotropic drug resistance protein 8) and *AtABCC1* (ATP-binding cassette sub-family C protein 1), *AtGSH1* (Glutathione 1) and *AtPCS1* (Phytochelatin synthase 1). Among them, AtIRT1 and AtNramp1 are the major players involved in Cd uptake in *A. thaliana* [29, 30]. AtHMA2 and AtHMA4 are responsible for transporting Cd from the roots to the shoots [31]. *AtPDR8* encodes efflux pump of Cd at the plasma membrane of cells [32]. AtABCC1 and AtHMA3 play a role in the detoxification of Cd by vacuolar sequestration [33, 34], while AtNramp3 and AtNramp4 are responsible for excreting Cd out of vacuoles [35]. AtGSH1 and AtPCS1 are responsible for synthesizing glutathione and phytochelatin, respectively, which can chelate Cd [36, 37].

**Fig. 7:**
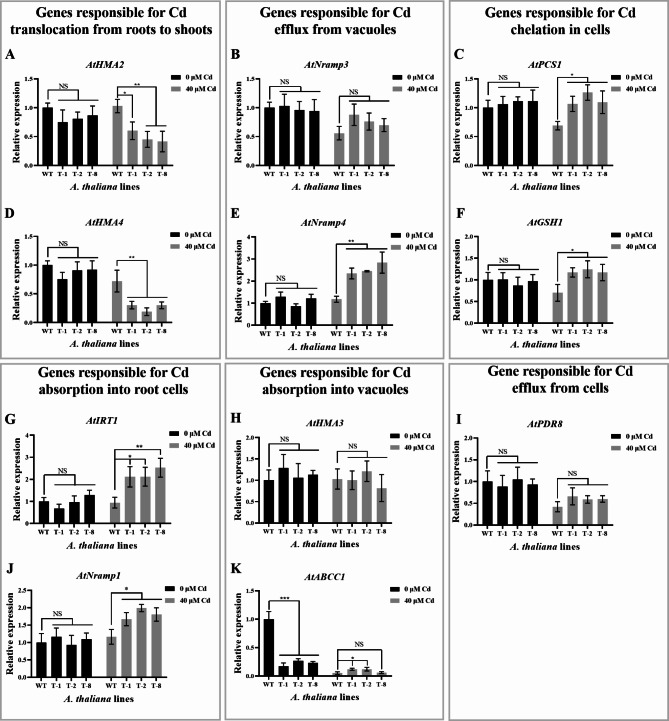
The expression of genes related to chelation, absorption and transport of Cd in *A. thaliana*. The expression of *AtHMA2* (**A**), *AtNramp3* (**B**), *AtPCS1* (**C**), *AtHMA4* (**D**), *AtNramp4* (**E**), *AtGSH1* (**F**), *AtIRT1* (**G**), *AtHMA3* (**H**), *AtPDR8* (**I**), *AtNramp1* (**J**) or *AtABCC1* (**K**) were analyzed using qRT-PCR. Results were calculated using 2^-ΔΔCT^ method with *AtUBQ10* as internal reference gene. The relative expression of each gene in the WT without Cd treatment was set as 1. Shown are mean ± standard deviation (SD) from three biological replicates. Asterisks indicate significant differences (Student’s t-test: NS means non-significant; *P < 0.05; **P < 0.01)

The results showed that, without Cd treatment, the expression of genes related with chelation, absorption and transport of Cd in both transgenic and WT *A. thaliana* plants was generally unaffected, only with the exception of the downregulation of *AtABCC1* (Fig. 7A-K). Interestingly, the expression of genes responsible for Cd uptake into root cells (*AtIRT1* and *AtNramp1*) was significantly upregulated in transgenic *A. thaliana* under Cd stress compared to the WT (Fig. 7G and J). Consequently, these results suggest that *OsHARBI1-1* may enhance Cd accumulation in *A. thaliana* by upregulating the expression of *AtIRT1* and *AtNramp1*. In addition, under Cd stress, the expression of *AtHMA2* and *AtHMA4* which are responsible for translocating Cd from the roots to the shoots, were significantly downregulated in transgenic *A. thaliana* compared to the WT (Fig. 7A and D). Conversely, the expression of *AtPCS1* and *AtGSH1*, which are responsible for chelating Cd within cells, showed a significant increase (Fig. 7C and F). These findings imply that *OsHARBI1-1* may enhance the tolerance of *A. thaliana* to Cd by suppressing the translocation of Cd from the roots to the shoots while simultaneously improving Cd chelating capacity.

### Expression of *OsHARBI1-1* affected the expression of various phytohormone related genes in *A. thaliana* under Cd stress

Phytohormones are reported to play important roles in regulating root development and combating Cd toxicity [3, 38]. Microarray data from RiceXpro revealed that the expression of *OsHARBI1-1* was induced by ABA and JA (Fig. S2). Additionally, previous studies have indicated that overexpression of *AtHARBI1-1* and *AtHARBI1-2* affects the levels of auxin in *A. thaliana* [26, 27]. Therefore, to further investigate the potential involvement of these phytohormones in Cd tolerance in transgenic *A. thaliana*, the expression of genes responsible for the biosynthesis and signaling transduction of auxin, ABA and JA were determined in *A. thaliana* [39–45]. The functions of these genes in phytohormone biosynthesis and signaling transduction are shown in Figure S5.

**Fig. 8:**
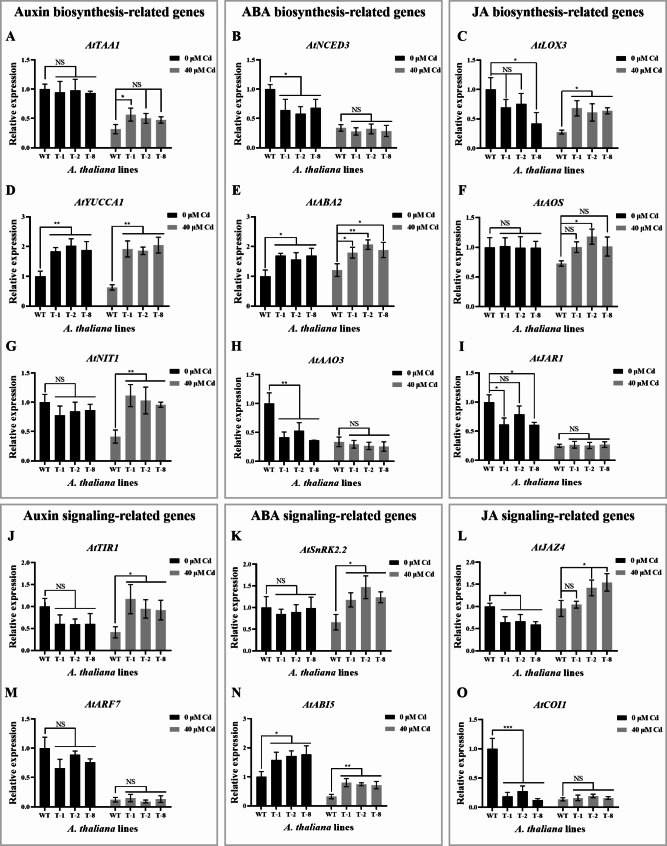
The expression of phytohormone-related genes in *A. thaliana*. The expression of *AtTAA1* (**A**), *AtNCED3* (**B**), *AtLOX3* (**C**), *AtYUCCA1* (**D**), *AtABA2* (**E**), *AtAOS* (**F**), *AtNIT1* (**G**), *AtAAO3* (**H**), *AtJAR1* (**I**), *AtTIR1* (**J**), *AtSnRK2.2* (**K**), *AtJAZ4* (**L**), *AtARF7* (**M**), *AtABI5* (**N**) or *AtCOI1* (**O**). Results were calculated using 2^-ΔΔCT^ method with *AtUBQ10* as internal reference gene. The relative expression of each gene in the WT without Cd treatment was set as 1. Shown are mean ± standard deviation (SD) from three biological replicates. Asterisks indicate significant differences (Student’s *t*-test: NS means non-significant; *P < 0.05; **P < 0.01)

The results showed that without Cd treatment, the expression of IAA (3-Indoleacetic acid)-related gene *AtYUCCA1* (Flavin monooxygenase 1) and ABA-related genes *AtABA2* (ABA deficient 2) and *AtABI5* (ABA insensitive 5) in transgenic *A. thaliana* were significantly increased, compared to those of the WT (Fig. 8D, E and N). Conversely, the expression levels of ABA-related genes *AtNCED3* (9-cis-epoxycarotenoid dioxygenase 3) and *AtAAO3* (Abscisic aldehyde oxidase 3) as well as JA-related genes *AtLOX3* (Lipoxygenase 3), *AtJAR1* (Jasmonate resistant 1), *AtJAZ4* (Jasmonate-zim-domain protein 4) and *AtCOI1* (Coronatine insensitive 1), were significantly reduced in transgenic *A. thaliana* compared to those of the WT (Fig. 8B, C, H, I, L and O). There were no significant differences in the expression of genes such as *AtTAA1* (Tryptophan aminotransferase 1), *AtNIT1* (Nitrilase 1), *AtTIR1* (Transport inhibitor response 1), *AtARF7* (Auxin response factor 7), *AtSnRK2.2* (Sucrose non-fermenting protein kinase 2.2) and *AtAOS* (Allene oxide synthase) between transgenic and the WT plants (Fig. 8A, F, G, J, K and M). However, under Cd treatment, the expression of numerous hormone-related genes in transgenic *A. thaliana* was significantly increased compared to that in the WT. These genes included IAA synthesis-related genes *AtTAA1*, *AtYUCCA1*, *AtNIT1* and *AtTIR1*, ABA-related genes *AtABA2*, *AtSnRK2.2* and *AtABI5* as well as JA-related genes *AtLOX3*, *AtAOS* and *AtJAZ4* (Fig. 8). Meanwhile, the expression of the other genes was not significantly different from those of the WT (Fig. 8). These findings indicate that *OsHARBI1-1* affects the expression of many phytohormone-related genes so as to influence both root growth and Cd resistance in *A. thaliana*.

## Discussion

With the wide-spread application of transcriptome sequencing technology, numerous rice Cd-responsive genes in rice have been investigated [17]. However, the functions of many of these genes remain largely unknown. In this study, 33 *HARBI1* genes in rice were identified (Fig. 1 and Table S2). However, bioinformatics analysis showed that only *HARBI1-1* and *HARBI1-2* of the *33 HARBI1* genes were induced by Cd stress and these two proteins were also structurally close to each other (Fig. 1). Notably, the expression of *OsHARBI1-1* in the roots shown in Fig. 2A-C contradicts the results presented in Fig. 1C. This consistency could be attributed to the variations in rice cultivars used for gene expression analysis, as well as cultivation conditions and rice seedling size [46, 47]. Our study has provided evidence that *OsHARBI1-1* could affect Cd tolerance in both yeast and plants, thus, it is of interest to explore the role of *OsHARBI1-2* in plant responses to Cd stress and determine whether there is functional redundancy between *OsHARBI1-1* and *OsHARBI1-2*.

The discovery of a number of cis-elements related to phytohormones (such as auxin, ABA, GA and MeJA) in the promoter of *OsHARBI1-1* was not unexpected (Fig. S1), implying that the expression of *OsHARBI1-1* may be regulated by phytohormones. This inference was further supported by the data from RiceXpro (Fig. S2). In this study, the root length of transgenic plants was significantly increased than that of the WT plants without Cd treatment (Fig. 5B and C). Plant root growth is regulated by phytohormones such as ABA, JA or IAA [48, 49]. Expression of *OsHARBI1-1* influenced the expression of multiple hormone-related genes in *A. thaliana*, suggesting its impact on hormone homeostasis and potentially contribution to the root growth in transgenic *A. thaliana*. In addition, phytohormones play a role in Cd tolerance in plants [50]. For instance, the absorption of Cd regulated by ABI5-MYB49 interaction is mediated by ABA signaling [51]. Moreover, auxin reduces the toxicity of Cd by stimulating the activity of antioxidant enzymes, whereas Cd treatment induces JA biosynthesis, thereby reducing Cd toxicity [40, 44]. The expression of *OsHARBI1-1* significantly upregulated most of the genes under Cd stress among the examined hormone-related genes. This provides evidence for the crucial role of *OsHARBI1-1* in regulating Cd tolerance by activating the biosynthesis and signaling pathways of phytohormones.

Harbinger transposase has the distinctive ability to move within genomes due to it structural features such as terminal inverted repeats, flanking target site duplication and HTH motif [19, 22]. However, as OsHARBI1-1 protein lacks the typical characteristics of the transposon described above, it may be a protein with novel functions evolved from the nuclease component of Harbinger transposase. This phenomenon has been reported previously [21–23]. Most of the *HARBI1* genes reported so far are related to plant growth and development, or stress resistance [21–27]. However, how transposons acquire novel functions during plant evolution remains to be further explored.

The functions of proteins are usually closely linked to their localization [21, 22]. In this study, the subcellular localization of OsHARBI1-1 was observed in nucleus in both tobacco and yeast cells (Fig. 4), further supporting the conclusion that HARBI1 protein performs its functions in the nucleus [21–25, 52]. Meanwhile, the nuclear localization of OsHARBI1-1 also suggests that OsHARBI1-1 protein may not have a direct effect of binding or transporting Cd to resist Cd stress. Furthermore, in human cells, the Myb-related transcriptional regulator interacts with HARBI1 and promote the nuclear import of HARBI1 [52]. Interestingly, the MYB binding site was also found in the promoter region of *OsHARBI1-1* (Fig. S1). Therefore, we cannot exclude the possibility that OsHARBI1-1 may regulate the tolerance of yeast or *A. thaliana* by sensing and passing the Cd signal to other regulators such as transcription factors. In addition, several studies have reported that another protein, HARBI2, also domesticated from Harbinger transposase, interacts with HARBI1 in the nucleus and is involved in the epigenetic mechanisms as a component of histone modification complexes [21–23]. Thus, identification of OsHARBI1-1 interacting proteins and their functions, particularly transcription factors like Myb, may provide the insight into the functions of *OsHARBI1-1* in response to heavy metal stress.

Several recent studies have shown that HARBI1 protein, as one of the components of the complex that mediates histone modification, may be involved in the regulation of plant stress responses such as cold and salt stress [23, 24, 53]. But whether this epigenetic machinery is responsible or partially responsible for HARBI1 mediated Cd response in plants remains unclear. Therefore, the roles of HARBI1 in histone modification mechanism deserves further clarification.

For plants, one of the strategies to deal with Cd stress is to reduce the toxicity of Cd by limiting the accumulation of Cd or chelating Cd with metal chelators within cells [12]. It was observed that the expression of *OsHARBI1-1* increased Cd content in both roots and whole seedlings of *A. thaliana* (Fig. 6A and B). This result might be attributed to the upregulation of Cd absorption-related genes *AtIRT1* and *AtNramp1* by *OsHARBI1-1*, leading to enhanced Cd uptake in *A. thaliana* [29, 30]. As multicellular organisms, plants have evolved more intricate mechanisms for Cd detoxification, and the varying ability of different tissues, organs or even cells to accumulate Cd is considered as an important factor for plants to tolerate Cd stress. At the organ level, plants tend to accumulate heavy metals in the roots, while at the cellular level, heavy metals are often sequestered within the vacuoles [54]. AtHMA2 and AtHMA2 serve as the primary transporters responsible for Cd translocation from the roots to the shoots in *A. thaliana* [31]. The expression of *OsHARBI1-1* resulted in significant downregulation in the expression of *AtHMA2* and *AtHMA2* (Fig. 8A and C), as well as a decrease in the Cd content in the shoots (Fig. 6C), suggesting a potential mechanism by which *OsHARBI1-1* enhances Cd tolerance in *A. thaliana* by inhibiting Cd translocation from the roots to the shoots. Within cells, *OsHARBI1-1* may act by chelating Cd to mitigate Cd toxicity, rather than sequestering Cd in vacuoles. This deduction was supported by the fact that *OsHARBI1-1* did not affect the expression of *AtHMA3* and *AtABCC1*, which are involved in vacuolar Cd transportation, but instead promoted the expression of *AtPCS1* and *AtGSH1*, which are related with Cd chelation (Fig. 8C, F, H and K) [32, 33, 36, 37]. The possible mechanism by which *OsHARBI1-1* enhances Cd tolerance in *A. thaliana* was shown in Fig. S6.

Expression of *OsHARBI1-1* enhanced Cd tolerance in yeast and *A. thaliana*, while a different situation was observed for Cd accumulation. As multicellular organisms, plants have evolved more intricate mechanisms for Cd detoxification, and the varying ability of different organs, tissues, or even cells to accumulate Cd is considered as an important factor for plants to tolerate Cd stress [55]. At the organ level, plants tend to accumulate heavy metals in the roots, while at the cellular level, heavy metals are often sequestered in the vacuoles [55]. Therefore, the concentration of Cd in the yeast cells showed a tendency to differ from that in plants when determining the concentration of Cd in organisms due to the yeast is markedly different from plants in either cell organization (unicellular or multicellular) or cell structure. Another possible explanation could be the differences of Cd accumulation mechanisms mediated by chelation, transportation in yeast and in *A. thaliana*. In this study, the affected gene expression of Cd absorption, transport or chelation-related genes, as well as affected translocation of Cd by the expression of *OsHARBI1-1* suggests that *OsHARBI1-1* may improve Cd tolerance in *A. thaliana* by reducing the Cd transport from the roots to the shoots and enhancing Cd chelation ability in plants (Figs. 6B-C and 7). Furthermore, previous studies have shown that auxin promotes the fixation of Cd by cell walls, thereby improving Cd tolerance in *A. thaliana* [56]. In this study, the significant upregulation of auxin biosynthesis-related genes was observed in transgenic *A. thaliana* under Cd stress (Fig. 8A, D and G), suggesting a potential auxin-mediated mechanism in the response of *A. thaliana* to Cd stress. However, thus far, no auxin-mediated mechanism has not been found to protect yeast from Cd toxicity, which also may explain the divergent Cd content between transgenic yeast and transgenic *A. thaliana*.

Antioxidant enzymes present in plants are effective in reducing oxidative damage caused by exposure to Cd [12, 54]. However, they also exert other functions such as regulation of plant root growth and hormone signaling which is implied by the enhanced activity of POD by exogenous auxin [57, 58]. Interestingly, in this study, the activity of POD was significantly increased regardless of Cd treatment, and the expression of auxin biosynthesis genes was significantly upregulated as well (Figs. 6E and 8A, D and G). Studies have shown that auxin may promote POD activity in plants [57, 59]. Therefore, the increased expression of auxin biosynthesis and responding genes and increased POD activity which were resulted from *OsHARBI1-1*, contribut to the promotion of root growth and Cd resistance in transgenic *A. thaliana*.

## Conclusions

In this study, the function of rice gene *OsHARIB1-1* under Cd stress was elucidated through both bioinformatics analysis and functional analysis in yeast and *A. thaliana*. According to the bioinformatics analysis results, *OsHARBI1-1* might play an essential role in response to abiotic stress via plant hormone signal transduction. As a Cd-responsive gene encoding a nuclear localization protein, *OsHARBI1-1* significantly improved the tolerance of Cd in yeast and *A. thaliana*. Our results also suggest that *OsHARBI1-1* may improve Cd tolerance in *A. thaliana* by reduceing translocation of Cd from the roots to the shoots. Furthermore, the affected expression of genes involved in phytohormone signaling, Cd absorption, transport, and chelation, coupled with the upregulation of POD activity, might represent the mechanisms by which *OsHARBI1-1* affects the tolerance of plants to Cd stress. In conclusion, this study proposes *OsHARBI1-1* as a novel candidate that functions in Cd stress response in plants.

## Materials and methods

### Bioinformatics analysis of *OsHARBI1* family

The protein sequence of OsHARBI1-1 and its families were obtained online via Rice Genome Annotation Project (http://rice.plantbiology.msu.edu) and The Rice Annotation Project Database (https://rapdb.dna.affrc.go.jp/). The domain and three-dimensional structure of OsHARBI1-1 protein was predicted by using Uniprot (https://www.uniprot.org/) and pfam (https://pfam.xfam.org/). PlantCARE online program (http://bioinformatics.psb.ugent.be/webtools/plantcare/html/) was used to analyze the promoters of *OsHARBI1* genes. For phylogenetic analysis, the phylogenetic tree was constructed by using MEGA7.0 software and Neighbor-Joining (NJ) method (Number of bootstrap replications was set to 1000) [60]. For analysis of *OsHARBI1* genes expression patterns under Cd stress, transcriptome data from Rice Expression Database (http://expression.ic4r.org/) were used to assess the expression profile of these *OsHARBI1* genes under Cd stress. The expression profiles of these *OsHARBI1* genes were evaluated by subjecting the samples to a 50 µM Cd treatment of 10-day-old seedlings of the rice cultivar Nipponbare [61]. The data of *OsHARBI1-1* expression pattern under phytohormone treatments (ABA, GA, IAA, BL (Brassinolide), tZ (trans-zeatin) and JA) was obtained from RiceXPro (https://ricexpro.dna.affrc.go.jp/). The project number for the data is RXP_1000.

### Plant growth conditions and cd treatment

Wild-type (WT) rice (*Oryza sativa*) seeds were soaked in deionized water and incubated for 48 h at 37℃ with shaking at 200 rpm. Subsequently, the germinated seeds were grown at 30℃ with a 16-h-light/8-h-dark cycle. For hydroponic culture, 5-day-old seedlings were used for 25, 50 or 100 µM Cd stress treatment, respectively. After Cd treatment with time gradients (1, 6 and 12 h), the shoots and roots of rice plants were sampled and snap-frozen in liquid nitrogen, and then stored at − 80 °C for subsequent analysis. Plants were sourced as follows: *A. thaliana* (Col-0) and rice (*Oryza sativa* ‘Dongjin’) was obtained and verified by A/Prof Ji Chen after cultivation at the Chengdu Campus, College of Agronomy, Sichuan Agricultural University, China. It was verified by Prof Ji Chen from her seed stock registrar and confirmed by visual examination of plants that are grown for seed stocks.

### RNA extraction and quantitative real-time PCR (qRT-PCR) analysis

Total RNA was extracted from *A. thaliana* and the roots and shoots of rice by using the RNA Extraction Kit (Aidlab Biotechnologies Co. Ltd, Beijing, China). According to the manufacturer’s instructions, the RevertAid First Strand cDNA Synthesis Kit (Thermo fisher, USA) was used to reverse-transcribed RNA (1 µg) into complementary DNA (cDNA). Three independent replicates of qRT-PCR reactions were performed for each sample by using 2 × T5 Fast qPCR Mix Kit (Aidlab Biotechnologies Co. Ltd, Beijing, China). The qRT-PCR reactions were carried out by using qTOWER3G IVD real-time PCR System (Applied Biosystems, Jena, Germany). The primers for qRT-PCR were listed in Table S1.

### Observation of the subcellular localization of OsHARBI1-1-eGFP protein

For subcellular localization analysis of OsHARBI1-1 in yeast cells, *pYES2-eGFP* or *pYES2-OsHARBI1-1-eGFP* recombinant vector was constructed by using *pYES2* expression vector as backbone and the gene expression of *OsHARBI1-1-eGFP* or *eGFP* was driven by *GAL1* promoter. These two recombinant vectors were transferred into yeast strain *BY4743* (*MATa/α his3Δ1/his3Δ1 leu2Δ0/leu2Δ0 LYS2/lys2Δ0 met15Δ0/MET15 ura3Δ0/ura3Δ0*). SD-Ura medium containing 2% galactose [w/v] was used for the induction of expression of eGFP proteins and chimeric proteins of OsHARBI-1 with eGFP. After 24 h of incubation, the fluorescence emitted by eGFP or OsHARBI1-1-eGFP was examined under a confocal microscope (Nikon A1 i90, LSCM, Japan). And for subcellular localization analysis of OsHARBI1-1 in tobacco cells, fragments of the coding sequences of *OsHARBI1-1* and *eGFP* were ligated to the binary vector *pHB*, and the gene expression of *OsHARBI1-1-eGFP* or *eGFP* was driven by CaMV *35 S* promoter. The vectors were transferred into *Agrobacterium tumefaciens* strain *GV3101*, followed by the infiltration into tobacco leaves by using a needleless syringe [62]. The nucleus staining was conducted with DAPI by dipping the tobacco leaves into DAPI solution for at least 30 min before fluorescence observation. The fluorescence was observed as described above.

### Functional analysis of *OsHARBI1-1* in yeast

The recombinant vector *pYES2-OsHARBI1-1* and the empty vector (as a negative control) were transformed into yeast mutant strains [*∆ycf1-BY4741* (*MATa his3∆1 leu2∆0 met15∆0 ura3∆0 YDR135c-kanMX4*)] by using the polyethylene glycol (PEG)-lithium acetate-based transformation method [63].

The yeast single colonies containing the recombinant vector *pYES2-OsHARBI1-1* and the empty vector were selected on SD-Ura medium plates and grown in the liquid SD-Ura medium at 30℃. After 24 h incubation, the liquid medium containing yeast cells was diluted for 10 folds with fresh liquid SD-Ura medium and the cells were incubated for another 2–3 h. When the OD600 values reached 0.5–0.8, the yeast cells were subjected to collection by centrifugation and concentrated to OD600 = 1 with sterile water. Subsequently, 5 µL of yeast cell fluid diluted in gradient (OD600 of 1.0, 0.1, 0.01 and 0.001) were spotted onto the surfaces of SD-Ura medium plates containing 2% galactose, 25 or 75 µM CdCl_2_ and incubated at 30 °C for 3 days. For liquid culture, the yeast cells were diluted with 20 mL liquid SD medium (with 2% galactose) to an OD600 of 0.1 in 50 mL flasks containing 0 and 25 µM CdCl_2_, respectively. The flasks were incubated on a rotary shaker at 200 rpm at 30 °C, and OD600 values were measured after 24 h.

### Functional analysis of *OsHARBI1-1* in *A. thaliana* under Cd stress

For expression of *OsHARBI1-1* in *A. thaliana*, the CDS sequence of *OsHARBI1-1* was amplified and ligated into the binary vector *pHB*, and the gene expression of *OsHARBI1-1* was driven by CaMV *35 S* promoter. Afterwards, the recombinant vectors were transferred into the *Agrobacterium tumefaciens* strain *GV3101* which was used to infect *A. thaliana* plants by using the floral dipping method [24]. Three independent lines were selected from T2 generation with high *OsHARBI1-1* relative expression by using qRT-PCR for all subsequent experiments.

For Cd treatment of *A. thaliana*, after the seeds of transgenic lines (T-2, T-8 and T-1) and WT were sterilized with 75% alcohol (added with 0.05% Triton X-100) and rinsed with sterile water, these seeds were placed on the surface of 1/2 MS solid media containing different concentrations of Cd (0 µM or 40 µM Cd), and then the media were placed in a 4℃ refrigerator for vernalization. After three days, the media were transferred to plant culture room for culture (23℃, 16-h-light/8-h-dark). The phenotypes of root length of *A. thaliana* seedlings were analyzed after two weeks of continuous cultivation.

### Determination of cd content

For determining Cd content in yeast cells and *A. thaliana* plants, the yeast cells were treated with 10 µM Cd for 12 h after overnight activation, while WT and transgenic *A. thaliana* lines (T-2, T-8 and T-1) were treated with 40 µM Cd for 2 weeks, followed by collection of yeast or *A. thaliana* samples. For determining Cd content, the roots, shoots or whole seedlings of WT and transgenic *A. thaliana* lines (T3-3, T3-5 and T3-6) were collected separately after 2 weeks of 40 µM Cd treatment. Finally, all the samples were washed for 3 times in deionized water and finally dried at 80 °C for 3 days. Following the procedures reported in the previous study, Cd concentration in the samples was quantified by using inductively coupled plasma-atomic emission spectrometry (ICP-AES) [64].

### Determination of antioxidant enzyme activities in *A. thaliana*

In order to assay the activities of antioxidant enzymes, fresh WT and transgenic *A. thaliana* seedling samples (~ 0.3 g) treated with 0 µM or 40 µM Cd for 2 weeks were ground and transferred to 2 mL centrifuge tubes. The extraction buffer (100 mM phosphate buffer of pH 7.6, 1 M EDTA, 0.3% Triton X-100, 2% polyvinyl polypyrrolidone) was added simultaneously for enzyme extraction. The determination of antioxidant enzyme activities for SOD (EC.1.15.1.1), POD (EC 1.11.1.7) and CAT (E.C. 1.11.1.6) were performed with some modifications according to previously published methods [65–67].

### Statistical analysis

All results in this study were obtained from three biological replications. All values in the charts were represented as “averages and standard deviations”. The data were statistically analyzed using the single-factor ANOVA method (Student’s t-test) (*P* ≤ 0.05–0.001). The statistical analysis was conducted by using Graphpad Prism 9 software (GraphPad Software, La Jolla, CA).

### Electronic supplementary material

Below is the link to the electronic supplementary material.

Supplementary Material 1

Supplementary Material 2

## Data Availability

statement. All the supporting data are included within the article and its additional files. Sequence data of *OsHARBI1* gene family can be found at The Rice Annotation Project Database (https://rapdb.dna.affrc.go.jp/) or Rice Genome Annotation Project (http://rice.plantbiology.msu.edu): *OsHARBI1-1* (*Os01g0582600*, *LOC_Os01g40070*), *OsHARBI1-2 (Os01g0186900*, *LOC_Os01g09220*), *OsHARBI1-3* (*Os01g0838900*, *LOC_Os01g62160*), *OsHARBI1-4* (*Os01g0894100*, *LOC_Os01g66930*), *OsHARBI1-5* (*Os02g0231600*, *LOC_Os02g13770*), *OsHARBI1-6* (*Os03g0608700*, *LOC_Os03g41200*), *OsHARBI1-7* (*Os03g0643050*, *None*), *OsHARBI1-8* (*Os04g0422900*, *LOC_Os04g34550*), *OsHARBI1-9 (Os04g0471100*, *LOC_Os04g39530*), *OsHARBI1-10* (*Os04g0644200*, *LOC_Os04g55130*), *OsHARBI1-11* (*Os05g0183900*, *LOC_Os05g09150*), *OsHARBI1-12* (*Os05g0184500*, *LOC_Os05g09210*), *OsHARBI1-13* (*Os05g0184901*, *LOC_Os05g09280*), *OsHARBI1-14* (*Os05g0252801*, *LOC_Os05g16400*), *OsHARBI1-15* (*Os05g0593000*, *LOC_Os05g51520*), *OsHARBI1-16* (*Os06g0164500*, *LOC_Os06g06910*), *OsHARBI1-17* (*Os06g0190950*, *LOC_Os06g09150*), *OsHARBI1-18* (*Os06g0226000*, *LOC_Os06g12170*), *OsHARBI1-19* (*Os06g0481850*, *None*), *OsHARBI1-20* (*Os06g0595433*, *LOC_Os06g39460*), *OsHARBI1-21* (*Os07g0116050*, *None*), *OsHARBI1-22* (*Os07g0175100*, *LOC_Os07g07880*), *OsHARBI1-23* (*Os08g0106900, LOC_Os08g01570*), *OsHARBI1-24* (*Os09g0122100*, *None*), *OsHARBI1-25* (*Os09g0292300*, *LOC_Os09g12050*), *OsHARBI1-26* (*Os10g0126100*, *LOC_Os10g03700*), *OsHARBI1-27* (*Os10g0460733*, *LOC_Os10g32290*), *OsHARBI1-28* (*Os10g0468250*, *None*), *OsHARBI1-29* (*Os11g0202600*, *LOC_Os11g09710*), *OsHARBI1-30* (*Os11g0577650*, *LOC_Os11g36920*), *OsHARBI1-31* (*Os11g0702700*, *LOC_Os11g47650*), *OsHARBI1-32* (*Os12g0299600*, *None*), *OsHARBI1-33* (*Os12g0500800*, *LOC_Os12g31660*). Transcriptome data of *OsHARBI1* gene family under Cd stress can be found at Rice Expression Database (http://expression.ic4r.org/): Project (DRP001141). Transcriptome data of *OsHARBI1-1* gene under phytohormones treatments can be found at RiceXPro (https://ricexpro.dna.affrc.go.jp/): Project (RXP_1000).

## Abbreviations

HARBI1: Harbinger Transposase Derived 1
Rice (Os): *Oryza sativa*
*A. thaliana * (At): *Arabidopsis thaliana*
Yeast: *Saccharomyces cerevisiae*
Tomato: *Solanum lycopersicum*
Md: *Malus domestica*
Tobacco: *Nicotiana benthamiana*
SOD: Superoxide dismutase
POD: Peroxidase
CAT: Catalase
GFP: Green fluorescence protein
MS: Murashige and Skoog medium
DAPI: 4’, 6-diamidino-2-phenylindole
PCR: Polymerase chain reaction
qRT-PCR: Quantitative real-time PCR
WT: Wild type
T: Transgenic
IAA: 3-Indoleacetic acid
MeJA: Jasmonic acid
GA: Gibberellin
ABA: Abscisic acid
JA: Jasmonic acid
BL: Brassinolide
tZ: Trans-zeatin
