# Supplementary material for: Expression of OsHARBI1-1 enhances the tolerance of Arabidopsis thaliana to cadmium
Source: BMC Plant Biol. 2023 Nov 11;23:556. doi: 10.1186/s12870-023-04540-0 (PMC10638780; doi:10.1186/s12870-023-04540-0)
Supplement: Supplementary file 1 — Supplementary Material 1 [file 12870_2023_4540_MOESM1_ESM.docx]

**Supplementary Tables**

The online version contains supplementary material available at https://doi

Table S1. Primers used in this study

| **Primer name** | **Primer sequence** | **Description** |
| --- | --- | --- |
| *OsHARBI1-1* 1F | ATGAGATCCACATCCGGC | Primers used for cloning *OsHARBI1-1* gene in plant expression vector (*pHB*) and yeast expression vector (*pYES2*) |
| *OsHARBI1-1* 1R | TCAGAAGAAGGTGGTTCC |  |
| *OsHARBI1-1* 2F | AGGTGCTGGAGAAGTCCATG | Primers used for *OsHARBI1-1* gene qRT-PCR analysis |
| *OsHARBI1-1* 2R | GTCTCGCAGATGTTGTGGAG |  |
| *OsUBQ*-F | ATCACGCTGGAGGTGGAGT | Rice reference gene primers for qRT-PCR analysis |
| *OsUBQ*-R | AGGCCTTCTGGTTGTAGACG |  |
| *AtUBQ10* F | AGAAGAAAAAGGCGAAAACCTC | *A. thaliana* reference gene primers for qRT-PCR analysis |
| *AtUBQ10* R | GGACTCTCTGAAGGACCCA |  |
| *AtABI5*-F | AGAGGGATAGCGAACGAGTCTAGTC | For qRT-PCR analysis |
| *AtABI5*-R | GTTCGGGTTTGGATTAGGTTTAGG |  |
| *AtNCED3*-F | ACAAGAACAAGGTCGCAAGATT | For qRT-PCR analysis |
| *AtNCED3*-R | CAGAGATGGAAGCAGAAGCAAT |  |
| *AtABA2*-F | TTGGGTAAAGTGGCATTGAT | For qRT-PCR analysis |
| *AtABA2*-R | GAAACCAACAAATGCATCCT |  |
| *AtSnRK2.2*-F | ATATGCCATCGGGATCTGAA | For qRT-PCR analysis |
| *AtSnRK2.2*-R | TTGGTTGGGAATGAAGAACAG |  |
| *AtAAO3*-F | AACCGCATGCGCACTAG | For qRT-PCR analysis |
| *AtAAO3*-R | GTCTTGCGGTTCAAAAACATCTT |  |
| *AtTIR1*-F | AGCGACGGTGATTAGGAGGT | For qRT-PCR analysis |
| *AtTIR1*-R | CATCCGTCAGGTACCAAATTAA |  |
| *AtARF7*-F | CCTCCGTCCAGCTCCCTT | For qRT-PCR analysis |
| *AtARF7*-R | CTTGTCGTGAAAACTGTGTCTCTT |  |
| *AtYUCCA1*-F | TCCGCATCGCTCCAAGGTTC | For qRT-PCR analysis |
| *AtYUCCA1*-R | GGAAGTATGGATCTGCGTTCTCACC |  |
| *AtTAA1*-F | AACGCTGCGACGGAGGATCG | For qRT-PCR analysis |
| *AtTAA1*-R | CGTGGACGGCGGCTTGACAA |  |
| *AtNIT1*-F | GGCGTTCATAACGAAGAAGGGCGTG | For qRT-PCR analysis |
| *AtNIT1*-R | TTCCTTCTCTATGGCTCCCATTACC |  |
| *AtLOX3*-F | TAACGATGCTGGTGTCCATC | For qRT-PCR analysis |
| *AtLOX3*-R | AATTGCCTGTGTGCAGCTAA |  |
| *AtAOS*-F | ATTAACGGAGCTTCCTAACGGCGA | For qRT-PCR analysis |
| *AtAOS*-R | TGGTGGCGAGGTTGTTTGTGATT |  |
| *AtJAR1*-F | ACGGCTCATCAAGTCCAGAAACA | For qRT-PCR analysis |
| *AtJAR1*-R | CAGGGTCAGTAGCGTTTCCA |  |
| *AtCOI1*-F | TTAGCCGTCGATTCCCGAAC | For qRT-PCR analysis |
| *AtCOI1*-R | TCGGAAGTGCACCGATTTGA |  |
| *AtJAZ4*-F | AGGTTCCAGTCAGCAAGACCA | For qRT-PCR analysis |
| *AtJAZ4*-R | GGTTCGGACATGGAGACTGTG |  |
| *AtABCC1*-F | GTTGACTGCGTCATTAGCCG | For qRT-PCR analysis |
| *AtABCC1*-R | AACTGAGAAGCAAACCCATCG |  |
| *AtIRT1*-F | TGGGTCTTGGCGGTTGTATC | For qRT-PCR analysis |
| *AtIRT1*-R | CCGAATGGTGTTGTTACCGC |  |
| *AtNRAMP1*-F | GTAGCCACTTCTCTTCTTATTTCAAG | For qRT-PCR analysis |
| *AtNRAMP1*-R | ACCTTTATTCTAGTACACTATCGAAAG |  |
| *AtPDR8*-F | CTACACTCTTCCTGAGAACCGAA | For qRT-PCR analysis |
| *AtPDR8*-R | TCATAGCCATCTCCGCAAACCC |  |
| *AtHMA3*-F | GCTACTATGAAGCGAGG | For qRT-PCR analysis |
| *AtHMA3*-R | GGTAACATGTAGACACGG |  |
| *AtNRAMP3*-F | CCGAGTCCAAGAAGCGCTAA | For qRT-PCR analysis |
| *AtNRAMP3*-R | CCGCGTTAACCAAACCGATG |  |
| *AtNRAMP4*-F | AACAATGATCGTGGCGCTTG | For qRT-PCR analysis |
| *AtNRAMP4*-R | AGTGGGATCACAGCGAAAGG |  |
| *AtHMA2*-F | GCTGAGGATTGCGTGGTT | For qRT-PCR analysis |
| *AtHMA2*-R | GATAAGTCCACAAGCACAAGCAC |  |
| *AtHMA4*-F | GAGCACGAATTGTTCCACGG | For qRT-PCR analysis |
| *AtHMA4*-R | AGGTCTCTACCTTGACGGCT |  |
| *AtGSH1*-F | GATGGTTTAGAGCGCAGAGG | For qRT-PCR analysis |
| *AtGSH1*-R | TACGCTTTGTCCCCATTCTC |  |
| *AtPCS1*-F | TCAGGGATCAAAGACCAAGC | For qRT-PCR analysis |
| *AtPCS1*-R | CCGTCGAAGATGCAATACCT |  |

Table S2. Summary of the characteristics of all members of *OsHARBI1*s

| Gene name | Locus | | ORF (bp) | Protein length (amino acid) |
| --- | --- | --- | --- | --- |
| *OsHARBI1-1* | *Os01g0582600* | *LOC_Os01g40070* | 1656 | 551 |
| *OsHARBI1-2* | *Os01g0186900* | *LOC_Os01g09220* | 909 | 302 |
| *OsHARBI1-3* | *Os01g0838900* | *LOC_Os01g62160* | 1191 | 396 |
| *OsHARBI1-4* | *Os01g0894100* | *LOC_Os01g66930* | 756 | 251 |
| *OsHARBI1-5* | *Os02g0231600* | *LOC_Os02g13770* | 1197 | 398 |
| *OsHARBI1-6* | *Os03g0608700* | *LOC_Os03g41200* | 834 | 277 |
| *OsHARBI1-7* | *Os03g0643050* | None | 702 | 233 |
| *OsHARBI1-8* | *Os04g0422900* | *LOC_Os04g34550* | 687 | 228 |
| *OsHARBI1-9* | *Os04g0471100* | *LOC_Os04g39530* | 600 | 199 |
| *OsHARBI1-10* | *Os04g0644200* | *LOC_Os04g55130* | 1644 | 547 |
| *OsHARBI1-11* | *Os05g0183900* | *LOC_Os05g09150* | 1452 | 483 |
| *OsHARBI1-12* | *Os05g0184500* | *LOC_Os05g09210* | 1065 | 354 |
| *OsHARBI1-13* | *Os05g0184901* | *LOC_Os05g09280* | 1407 | 468 |
| *OsHARBI1-14* | *Os05g0252801* | *LOC_Os05g16400* | 642 | 213 |
| *OsHARBI1-15* | *Os05g0593000* | *LOC_Os05g51520* | 1272 | 423 |
| *OsHARBI1-16* | *Os06g0164500* | *LOC_Os06g06910* | 1311 | 436 |
| *OsHARBI1-17* | *Os06g0190950* | *LOC_Os06g09150* | 600 | 199 |
| *OsHARBI1-18* | *Os06g0226000* | *LOC_Os06g12170* | 678 | 225 |
| *OsHARBI1-19* | *Os06g0481850* | None | 1068 | 355 |
| *OsHARBI1-20* | *Os06g0595433* | *LOC_Os06g39460* | 1188 | 395 |
| *OsHARBI1-21* | *Os07g0116050* | None | 1317 | 438 |
| *OsHARBI1-22* | *Os07g0175100* | *LOC_Os07g07880* | 1326 | 441 |
| *OsHARBI1-23* | *Os08g0106900* | *LOC_Os08g01570* | 1176 | 391 |
| *OsHARBI1-24* | *Os09g0122100* | None | 1296 | 431 |
| *OsHARBI1-25* | *Os09g0292300* | *LOC_Os09g12050* | 1206 | 401 |
| *OsHARBI1-26* | *Os10g0126100* | *LOC_Os10g03700* | 1485 | 494 |
| *OsHARBI1-27* | *Os10g0460733* | *LOC_Os10g32290* | 894 | 297 |
| *OsHARBI1-28* | *Os10g0468250* | None | 1296 | 431 |
| *OsHARBI1-29* | *Os11g0202600* | *LOC_Os11g09710* | 1245 | 414 |
| *OsHARBI1-30* | *Os11g0577650* | *LOC_Os11g36920* | 846 | 281 |
| *OsHARBI1-31* | *Os11g0702700* | *LOC_Os11g47650* | 1176 | 391 |
| *OsHARBI1-32* | *Os12g0299600* | None | 1188 | 395 |
| *OsHARBI1-33* | *Os12g0500800* | *LOC_Os12g31660* | 756 | 251 |
