# Supplementary material for: Expression of OsHARBI1-1 enhances the tolerance of Arabidopsis thaliana to cadmium
Source: BMC Plant Biol. 2023 Nov 11;23:556. doi: 10.1186/s12870-023-04540-0 (PMC10638780; doi:10.1186/s12870-023-04540-0)
Supplement: Supplementary file 2 — Supplementary Material 2 [file 12870_2023_4540_MOESM2_ESM.docx]

**Supplementary Figures**

The online version contains supplementary material available at https://doi


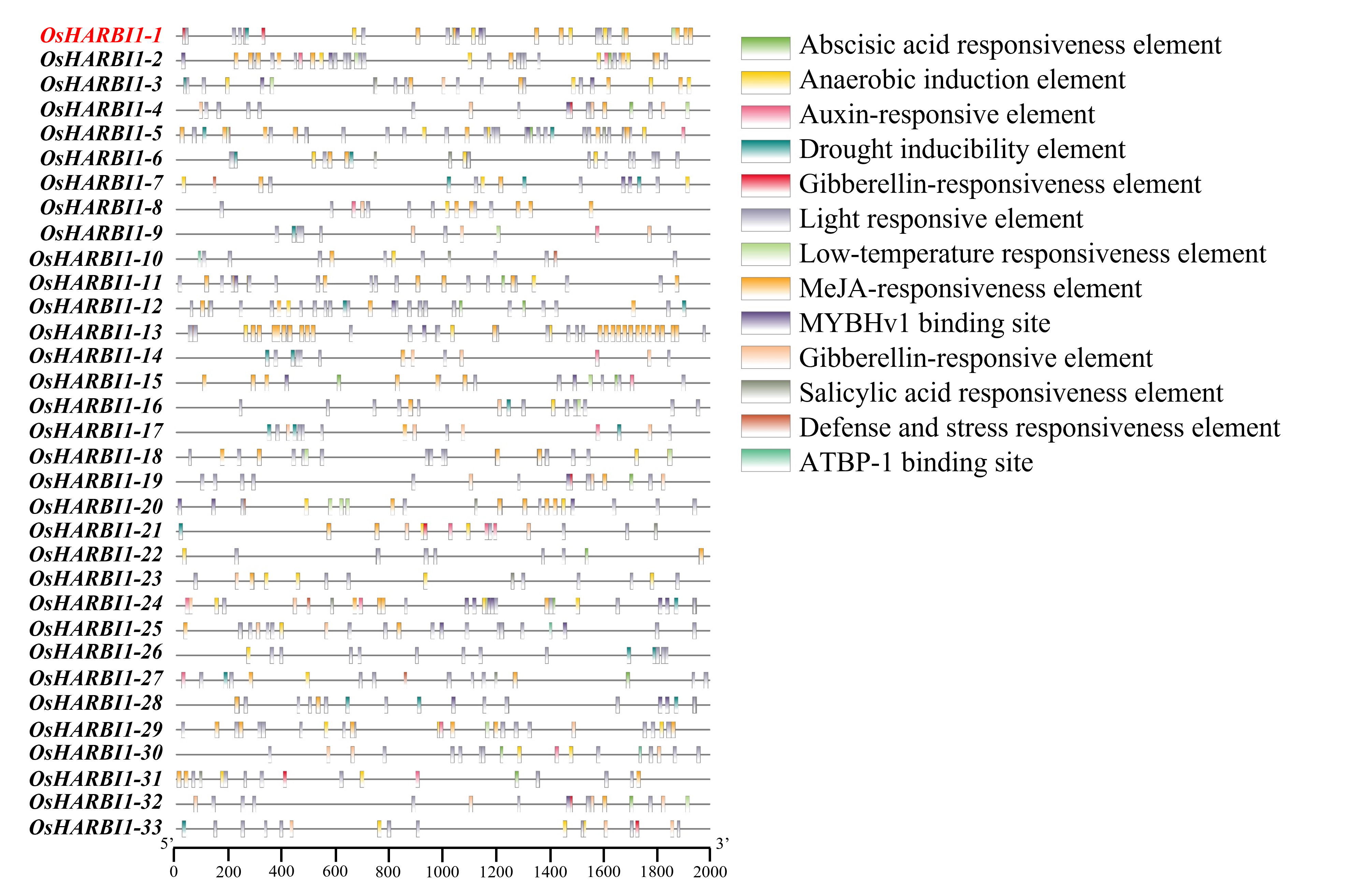


**Fig. S1.** Analysis of cis-regulatory elements of *OsHARBI1* family. The results were visualized by using TBtools software.


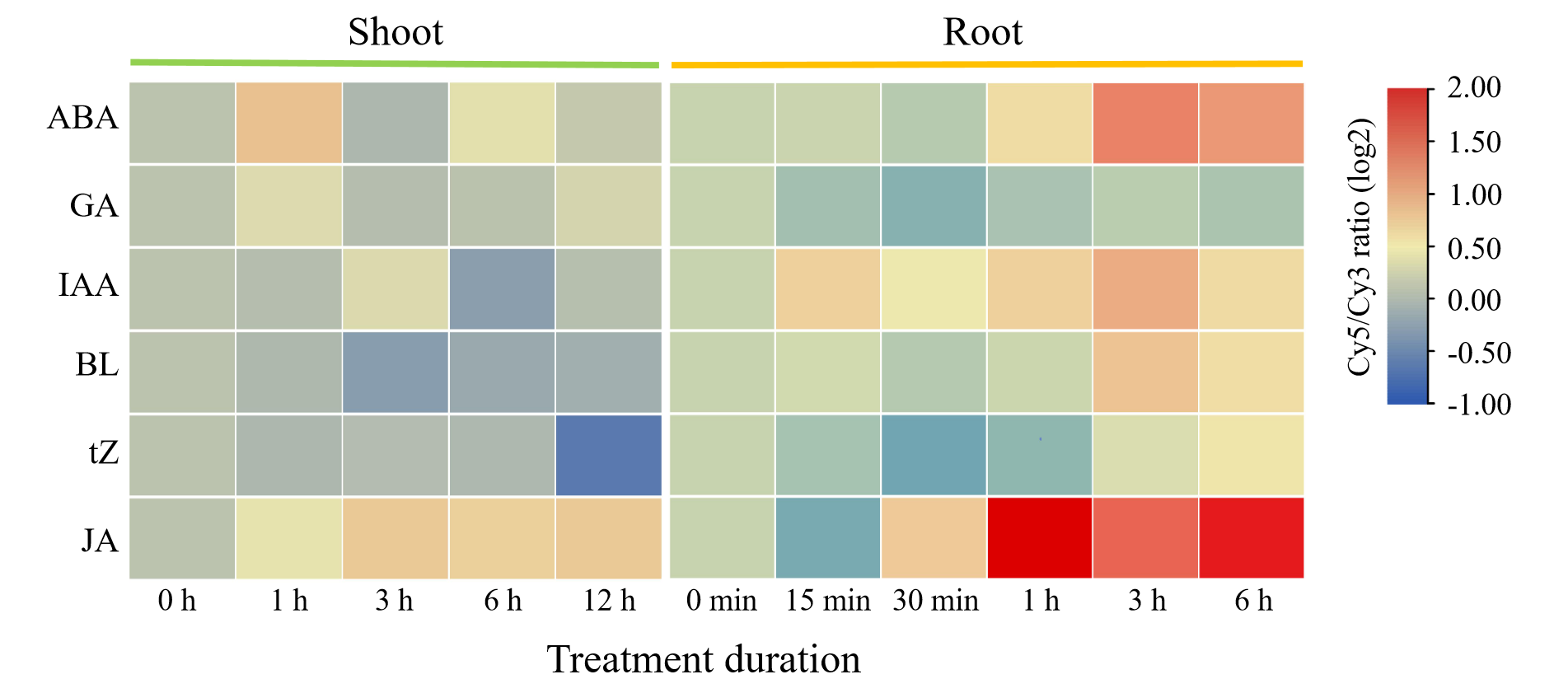


**Fig. S2.** Expression profiles of Os*HARBI1-1* gene under phytohormone treatments. The results used in the figure were retrieved from the Rice Expression Profile Database and were visualized in heat map format by using TBtools software. ABA (Abscisic acid), GA (Gibberellic acid), IAA (Indole-3-acetic acid), BL (Brassinolide), tZ (trans-zeatin), and JA (Jasmonic acid). The detail information of methods and materials used can be searched through the RiceXPro websites.


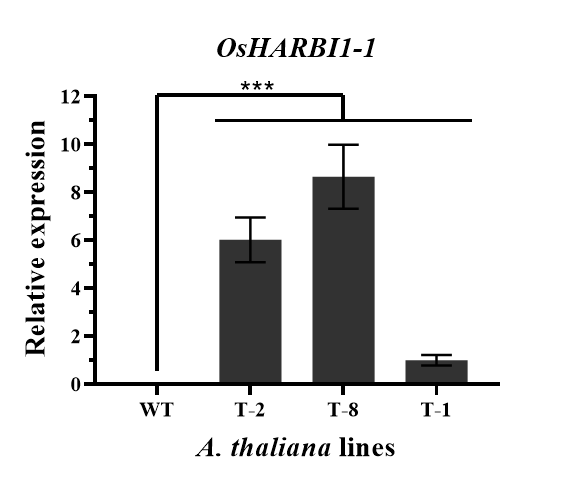


**Fig. S3.** Expression of *OsHARBI1-1* was analyzed in transgenic *A. thaliana* lines (T-1, T-2 and T-8) by qRT-PCR. *Ubiquitin* gene was used as an internal control. Each experiment with three biological replicates. Shown are mean ± standard deviation (SD) from three biological replicates. Asterisks indicate significant differences between transgenic *A. thaliana* lines and WT (Student’s t-test: ****P* < 0.001).


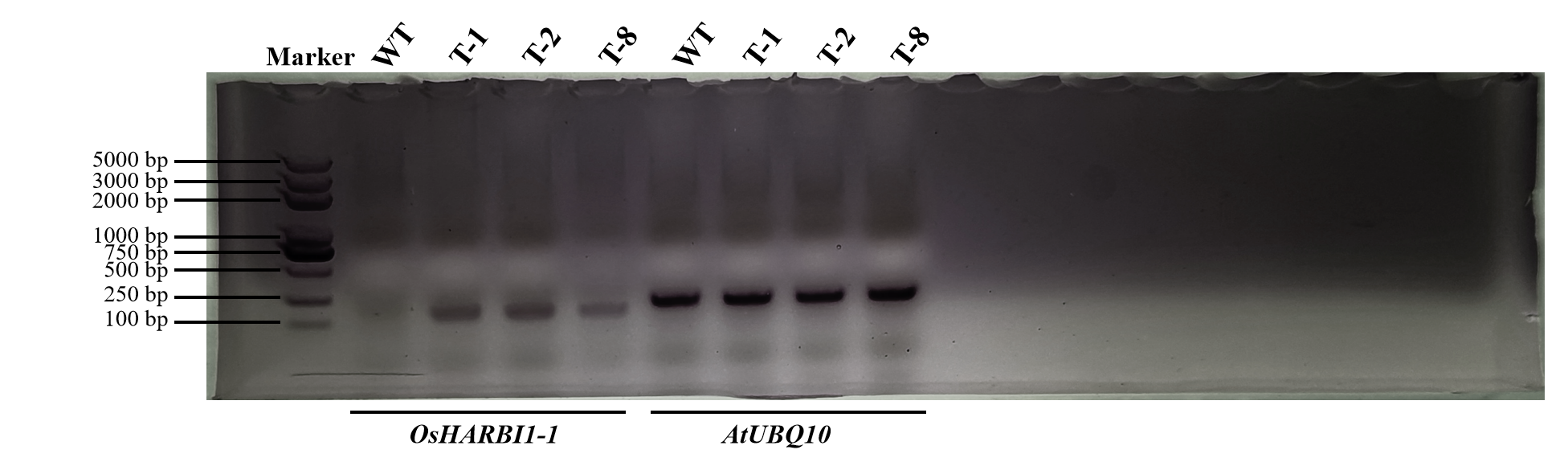


**Fig. S4.** Full-length gel and original image for figure 5. Uncropped and unexposed agarose gel electrophoresis results of *OsHARBI1-1* gene and *Ubiquitin* gene in WT and transgenic *A.* *thaliana* lines (T-2, T-8 and T-1). DNA was stained with GoldView.


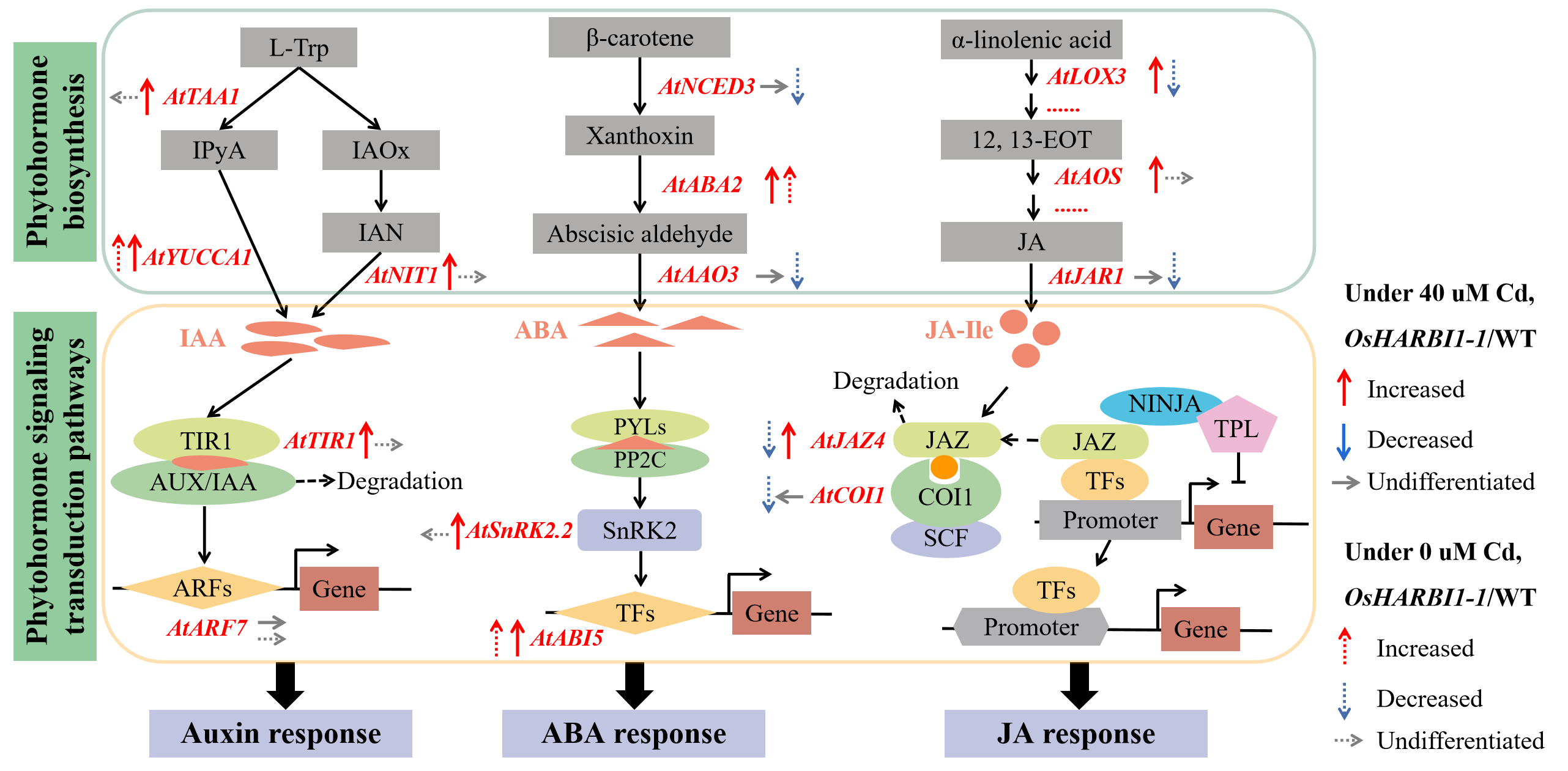


**Fig. S5** The effect of the expression of *OsHARBI1-1* on the expression of genes related with phytohormone biosynthesis and signaling. The solid arrows in red, blue, and gray represent increased, decreased, and undifferentiated expression of hormone-related genes in transgenic *A. thaliana*, compared to that of the WT under Cd stress. The corresponding dashed arrows represent change patterns in the expression of these genes without Cd stress. L-Trp (L-Tryptophan); IPyA (Indole-3-pyruvic acid); IAOx (Indole-3-acetaldoxime); IAN (Indole-3-acetonitrile); AUX/IAA (Auxin/Indole-3-acetic acid); PYLs (PYR1-like proteins); PP2C (Protein phosphatase 2C); 12,13-EOT (12,13(S)-epoxy-octadecatrienoic acid); NINJA (Novel interactor of JAZ); TPL (TOPLESS protein); SCF (Skp1, Cullin and F-box proteins); TFs (Transcription factors).


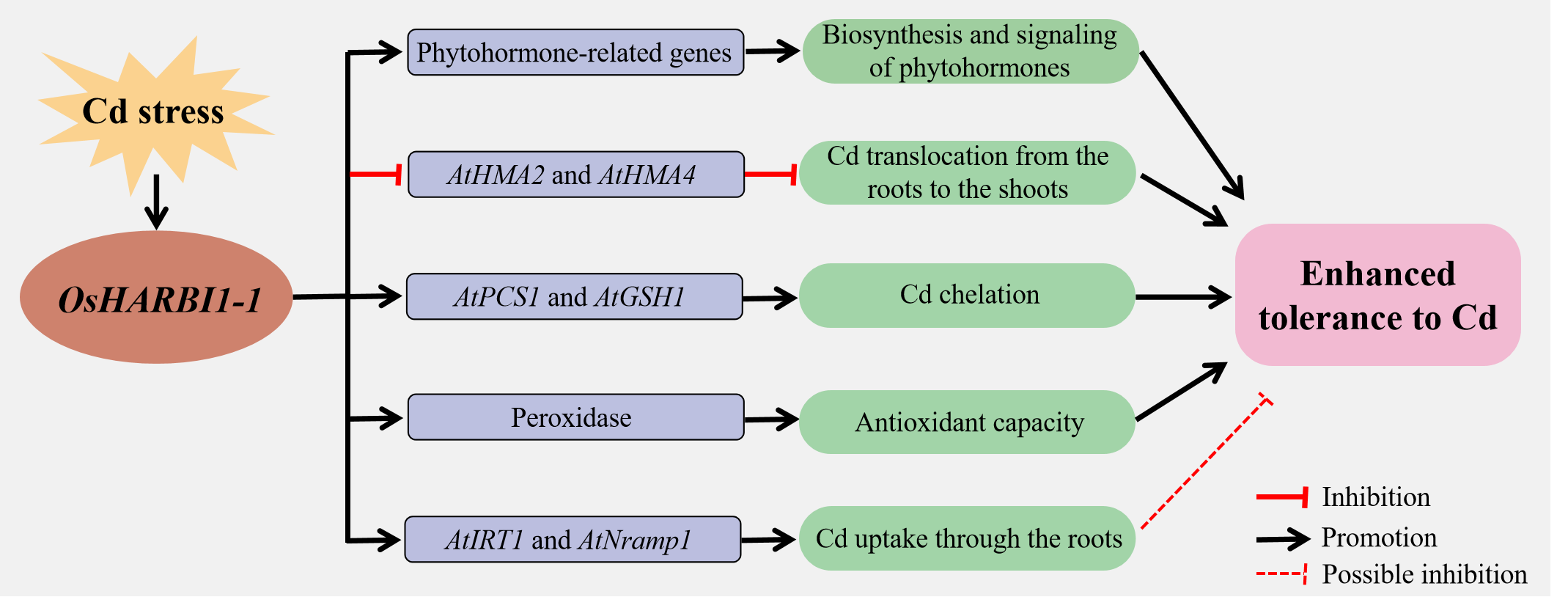


**Fig. S6** The schematics of potential mechanisms by which *OsHARBI1-1* enhances Cd tolerance in *A. thaliana* Black arrows indicate promotion; red T-arrows indicate inhibition, and red dashed T-arrows indicate possible inhibition.
